# Evaluation of Effects of the Human Immunodeficiency Virus on the Vestibular System by Using the Video Head Impulse Test

**DOI:** 10.5152/eurasianjmed.2022.20289

**Published:** 2022-06-01

**Authors:** Servet Karaca, M. Tayyar Kalcioglu, Fatma Sargin, Merve Torun Topçu, Lokman Uzun, Muhammet Tekin, Kamran Barin

**Affiliations:** 1 Departments of Otorhinolaryngology-Head and Neck Surgery, Istanbul Medeniyet University, Goztepe Training and Research Hospital, Istanbul, Turkey; 2 Infectious Diseases, Istanbul Medeniyet University, Goztepe Training and Research Hospital, Istanbul, Turkey; 3 Department of Audiology, School of Health Sciences, Istanbul Medeniyet University, Istanbul, Turkey; 4 Department of Otolaryngology-Head and Neck Surgery, The Ohio State University, Columbus, Ohio, USA

**Keywords:** AIDS, HIV, semicircular channels, vertigo, vestibulo-ocular reflex, vHIT

## Abstract

**Objective:** Some studies have suggested that the human immunodeficiency virus causes dizziness and other balance problems; however, the exact effects on the vestibular system in acute and chronic phases of the disease are not clear. In this study, we aimed to evaluate the effect of the human immunodeficiency virus on semicircular canals using a video head impulse test.

**Materials and Methods:** Seventy-two cases were included in the study. Twenty-six of the cases had positive human immunodeficiency virus RNA (group A) and 22 had negative human immunodeficiency virus RNA with positive anti-human immunodeficiency virus (group B) laboratory results. Twenty-four of the cases were healthy individuals (group C). The vestibular system was evaluated with a video head impulse test in all cases.

**Results:** In the evaluation of overt/covert saccades, a statistically significant difference was detected for the left posterior semicircular canal between group B and the other 2 groups. However, this was considered an incidental finding and not a clinically significant result. There was no other significant difference in the catch-up saccades for other canals. In addition, there was no statistically significant difference between the groups for the vestibulo-ocular reflex gain.

**Conclusion:** Although the human immunodeficiency virus has been reported to be vestibulotoxic in previous studies, we found that the video head impulse test findings were not affected in our patient groups. Because the video head impulse test is considered a high-frequency test of vestibulo-ocular reflex, it is possible that vestibular effects of the human immunodeficiency virus can be confined to low frequencies. It is also possible that HIV affects the central structures while sparing the peripheral vestibular pathways.

**Main Points**

- Human immunodeficiency virus (HIV) causes dizziness and other balance problems; however, the exact effects on the vestibular system in acute and chronic phases of the disease are not clear. In this study, we aimed to evaluate the effect of HIV on semicircular canals using a video head impulse test (vHIT).
- Although HIV has been reported to be vestibulotoxic in previous studies, we found that the vHIT findings were not affected in our patient groups.
- The results of this study indicated that the vestibular effects of HIV may be limited to lower frequencies. It is also possible for HIV to affect the central structures while protecting the peripheral vestibular pathways.

## Introduction

Vertigo is one of the frequently seen health problems. Besides the peripheral and central pathologies, vertigo may be caused by visual pathologies, metabolic problems, and cardiovascular disorders or by the side effects of many medications.^1-3^

While some studies have reported that human immunodeficiency virus (HIV) infection has an effect on the vestibular system,^4-6^ other studies have not found any effects.^7,8^ These conflicting results point to the need for further investigation of the vestibular system in HIV-infected patients.

The video head impulse test (vHIT) is a practical and non-invasive test that has been introduced in recent years for the evaluation of the vestibular system.^9^ The test was adapted from the clinical head impulse test, first described in 1988.^10^ Two decades later, in 2009, the vHIT system was first demonstrated to be a valid tool for testing the function of the horizontal^11,12^ and later, in 2013, the vertical semi-circular canals (SCCs).^13,14^ Hag et al^15^ in normative data studies in vertical semicircular canals found the vestibulo-ocular reflex (VOR) gain in right anterior, left posterior, left anterior, and right posterior canals to be 1.46 (0.32), 1.43 (0.37), 0.96 (0.19), 0.97 (0.19)/s, respectively. Mutlu et al^16^ found the VOR gain of right and left lateral canals to be 0.86 (0.1) and 0.93 (0.1)/s, respectively. Video head impulse test can help in differentiating causes of peripheral and central vertigo. It allows independent evaluation of anterior, posterior, and horizontal semicircular canals, which are components of the peripheral vestibular system.^17-19^ The aim of this study was to evaluate the effect of HIV infection on the VOR and SCCs in the acute and chronic phase of HIV infection.

## Materials and Methods

After receiving approval from the local ethical committee of İstanbul Medeniyet Goztepe Training and Research Hospital (approval date: February 10, 2015, Approval Number: 2015/0005), this research was performed between September 2015 and October 2016 as a collaborative study by Otorhinolaryngology and Head and Neck Surgery and Infectious Diseases and Clinical Microbiology Departments of the University Hospital, Istanbul Medeniyet University, Istanbul, Turkey. Informed consent was obtained from patients who participated in the investigation. The study was performed with 3 groups. Group A consisted of 30 patients who were in the acute stage of the disease with positive HIV RNA and had not received any treatment yet. Thirty patients in group B were in the chronic stage of the disease with negative HIV RNA and positive anti-HIV. They had received treatment for HIV infection. In group C, as the control group, there were 30 healthy volunteers who did not have any vestibular problem and had not been diagnosed with HIV and hence had negative HIV RNA and anti-HIV. Eighteen of 90 cases were excluded from the study because of failure such as focusing problems or touching the glasses with hands during the test, and 72 cases were included in the study. Twenty-six of these cases were in group A, while 22 were in group B and 24 in group C.

In all cases, vestibular system evaluation was performed with vHIT (GN Otometrics, ICS Impulse, Denmark). The patients were seated approximately 1 m from the wall. A visual target was placed at the eye level in front of the patient. Video head impulse test goggles were placed on the patient’s head and fastened tightly to prevent slippage artifacts. The examiner performed head impluses using high velocity head movements in the plane of each of the 3 SCCs. Patients with neck problems were excluded from the study. For the VOR gain, the normal range was considered to be values ≥0.8 for the lateral canal tests and ≥0.7 for the left vertical canal tests. The test results were evaluated by an experienced audiologist and an otorhinolaryngologist. The data were reviewed by the author (KB) who has experience with vHIT for the accuracy of VOR gain and for the presence of overt/covert saccade.

### Statistical Analysis

For statistical analysis, International Business Machines Statistical Package for the Social Sciences Software v.22 (IBM SPSS Corp.; Armonk, NY, USA) was used. By using Shapiro–Wilks test, the data were checked for normal distribution. Continuous variables were summarized by using average, standard deviation as well as frequency distributions. One-way analysis of variance was used to compare multiple group means with post hoc analysis such as Tukey’s honest significance test. For non-normally distributed data, Kruskal–Wallis test was used to identify the intergroup discrepancy. In addition, for qualitative data, chi-square, Fisher’s exact chi-square, and Fisher Hamilton test were used. The significance was accepted as *P* < .05.

## Results

Eighteen of 90 cases were excluded from the study because of the failure such as focusing problems or touching of the glasses with hands during the test, and 72 cases were included to the study. Twenty-six of these cases were in group A, while 22 in group B and 24 in group C. The age ranges of the cases in the groups A, B, and C were 19-47 (mean/standard deviation: 32.12 ± 7.93), 21-66 (39.64 ± 10.96), and 20-60 (36.42 ± 9.93), respectively (Table 1). There were 26 male patients in group A, 3 female and 19 male patients in group B, and 3 female and 21 male subjects in group C (Table 1). Two cases from group A, 1 from group B, and 2 from group C were exempted from the study because vHIT results were contaminated by artifacts. Therefore, the number of cases whose vHIT results were assessed was 24, 21, and 22 in groups A, B, and C, respectively.

### Group A

The level of HIV RNA was between 129 × 10^7^ and 1.6 × 10^7^ copy/mL (mean/SD: 8.9 × 10^4^ ± 1.4 × 10^4^). The CD4+ level of the patients was between 20 and 1102/mm^3^ (mean/SD: 349.38 ± 257.18) (Table 2).

The VOR gains for all of the canals are given in Table 2.

**Figure 1.**
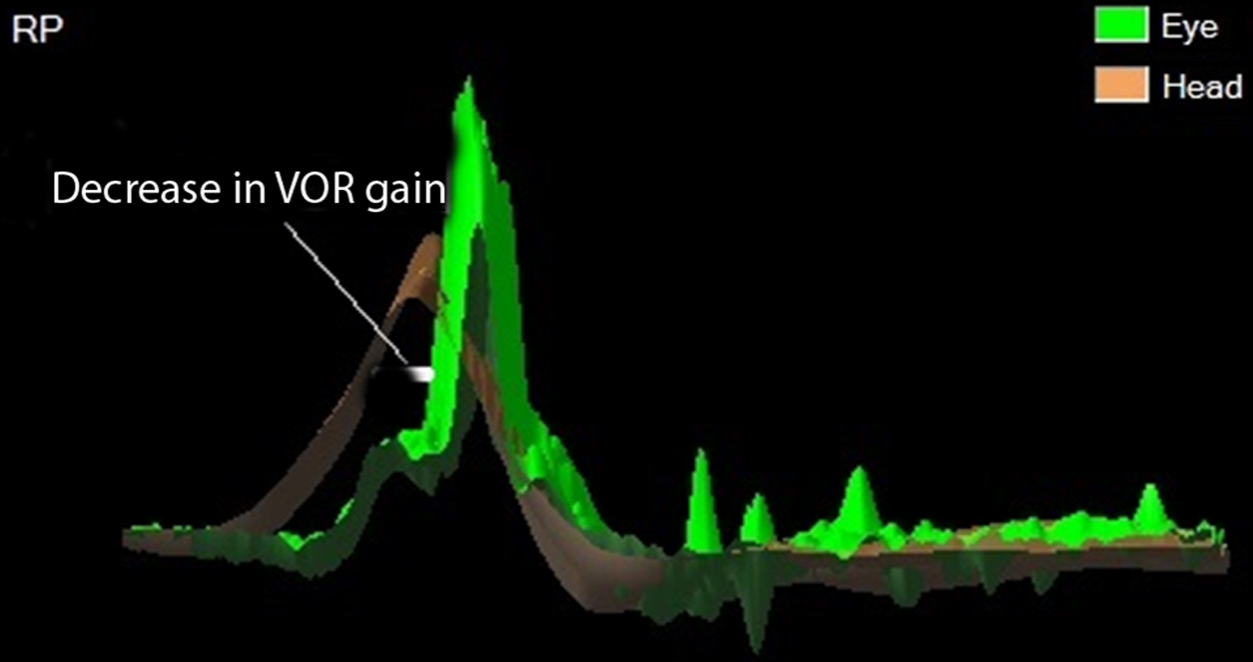
The 3D image of the decrease in vestibulo-ocular reflex gain in the right posterior semicircular canal.

In this group, no changes were observed in the VOR gain for the left lateral (LL) and right lateral (RL), while for left anterior (LA), right posterior (RP), left posterior (LP), and right anterior (RA), decreased VOR gains were seen in 12.5%, 12.5%, 12.5%, and 8.3% of the cases, respectively (Table 3) (Figure 1).

In this group, no overt/covert (O/C) saccade was seen in any of the cases (Table 4).

### Group B

The levels of HIV RNA were negative for all cases. The CD4+ levels were 207-1549/mm^3^ (average: 682.41 ± 382.84) (Table 2).

The VOR gain values of all the canals are given in Table 2.

In this group, while any decreased VOR gain was not detected in LL, RL, LA, and RA, a decrease in VOR gain was observed in 28.6% and 4.8% of the cases in RP and LP, respectively (Table 3).

In this group, overt or covert saccades (O/C) were not seen in LA and RA canals of the cases but 4.8%, 4.8%, 9.5%, and 14.3% of the cases had O/C in LL, RL, RP, and LP, respectively (Table 4).

### Group C

The levels of HIV RNA were negative for all cases. Since they were not infected with HIV, the level of CD4+ was not checked. The VOR gain values of all the canals are given in Table 2.

In this group, no decreased VOR gain was detected in RL and RA but decreased VOR gains were observed in 4.5%, 9.1%, 36.4%, and 13.6% of the cases in LL, LA, RP, and LP, respectively (Table 3).

**Figure 2.**
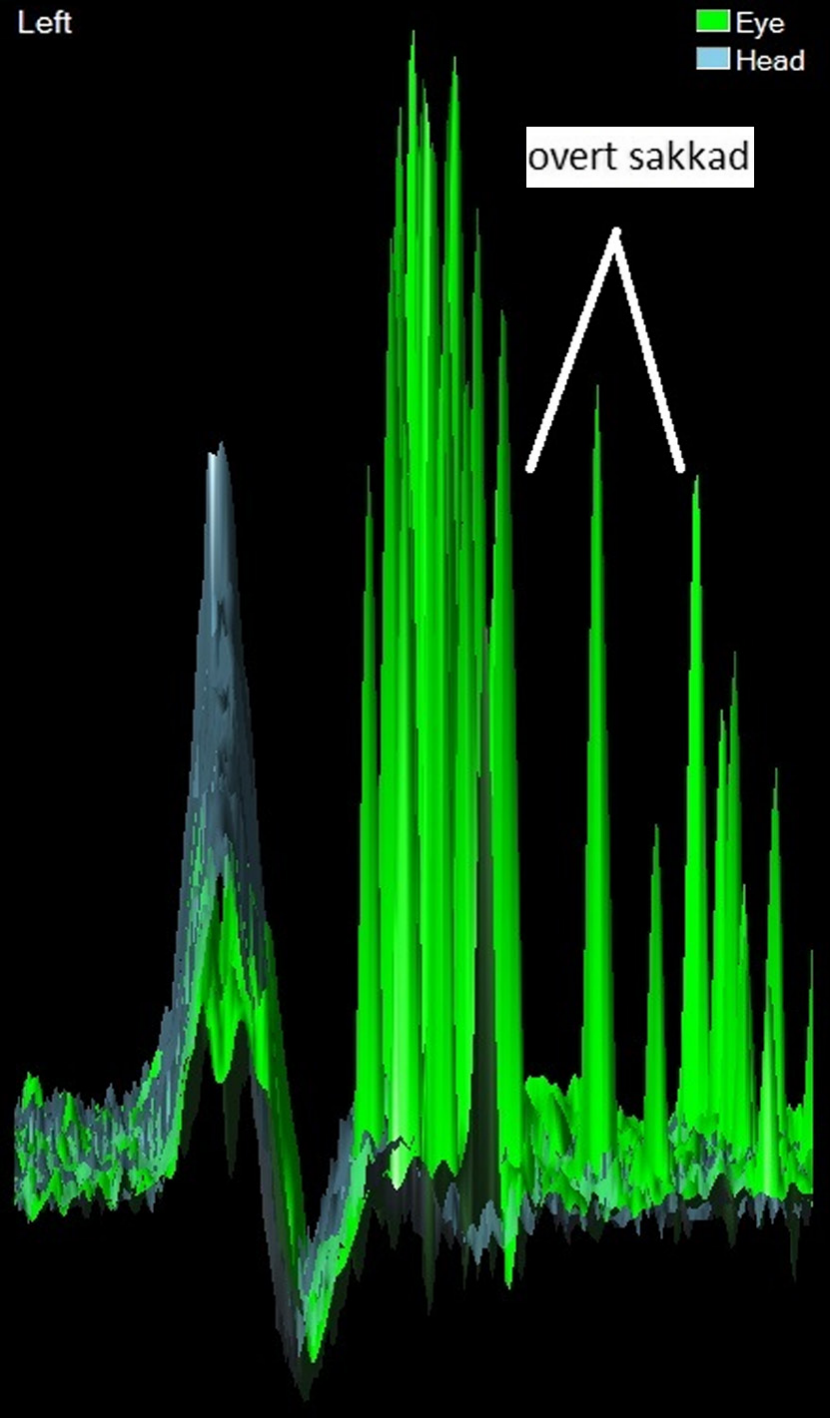
The 3D overt saccade image of the left lateral semicircular canal.

**Figure 3.**
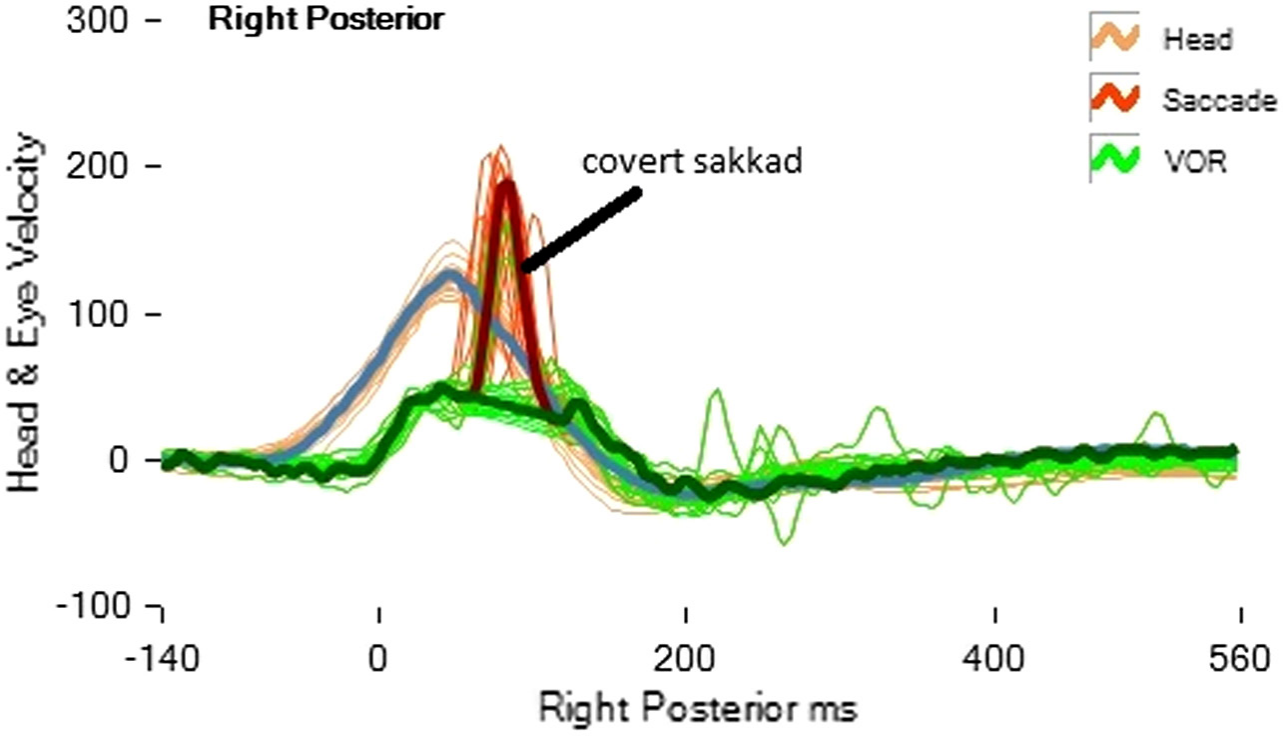
The 2D covert saccade image of the right posterior semicircular canal.

None of the cases had O/C in this group in LL, LA, LP, and RA, while 4.5% and 9.1% of the cases had O/C in RL and RP, respectively (Table 4) (Figures 2, 3).

When we compared the VOR gains of the groups, no statistically significant difference was seen between the groups (*P* > .05) (Table 5). When we focused on the O/C, group B had statistically significant differentiation at LP than the other groups (*P* = .028). Other canals did not have any significant difference when we compared the groups with each other (*P* > .05) (Table 6).

## Discussion

Human immunodeficiency virus and acquired immune deficiency syndrome (AIDS) affect millions of people directly or indirectly. Acquired immune deficiency syndrome is a multifaceted clinical entity that results in widespread clinical manifestations including head and neck findings [ 20]. The incidence of clinical symptoms in the head and neck region is reported to be between 40% and 90%.^21-26^ These symptoms may be due to the direct effect of HIV infection or opportunistic infections or toxic effects of used drugs.^27,28^

One of the effects of the AIDS on the inner ear is auditory pathology. Acquired immune deficiency syndrome-related auditory pathology includes regions of external and internal ear, cochlea, neural pathways, and central nervous system. Symptoms may involve 1 or more of these structures. Opportunistic infections involving external and middle ear, acute otitis externa, and otitis media with effusion can be seen.^29,30^ High-efficiency antiretroviral treatments and medical treatments for opportunistic infections can cause ototoxicity resulting in hearing loss, tinnitus, and hyperacusis.^31-33^ A direct effect of HIV on the neural pathways leading to neurological hearing disorders has also been reported.^31,34,35^ Although the exact incidence and prevalence of auditory disturbances is not known, up to 75% of adults with AIDS develop auditory pathologies.^36^ In the current study, none of HIV RNA+ individuals, HIV RNA−/anti-HIV+ individuals, and healthy volunteers had any hearing problems or tinnitus complaints. The presence of a hearing problem was only been questioned and no additional hearing assessment was conducted since the current study is directed only at vestibular problems.

There is a limited number of articles on the prevalence of vertigo in patients with AIDS.^4,5^ In a study, 9% of patients with AIDS were reported to have complained of vertigo, while another study reported numbers as high as 30%.^4,5^ More chronic and non-communicable symptoms, such as vestibular symptoms, are less reported because other life-threatening symptoms are at the forefront of AIDS patients.^4,5^ Although vestibular disorders have been reported in HIV-infected individuals, there are only a few reports of different types of vestibular involvement.^37^

Castello et al^7^ evaluated the vestibular system of 29 HIV individuals. In their studies, patients were classified according to the criteria of “Centers for Disease Control;” Stage II, III, and IV had asymptomatic patients, persistent generalized lymphadenopathies, and HIV-related complications, respectively. It was reported that in the caloric test, qualitative nystagmus eye movements were observed in 52% of the patients but no quantitative nystagmus was observed in any of the patients.^7^ Only the lateral SCC can be assessed by caloric test. This causes the superior and posterior SCC pathology to be overlooked. With vHIT, however, all SCCs can be evaluated in detail.

Hausler et al^6^ reported that central vestibular pathologies were detected by electronistagmography (ENG) in 22% of asymptomatic stage II patients, 50% of stage III patients, and 57% of stage IV patients. In another study conducted by Teggi et al^38^ with ENG, peripheral and central vestibular involvement was reported with a high prevalence. They reported abnormal peripheral and central vestibular symptoms in up to 40% in symptomatic patients in the advanced stage. There are 6 main tests on the ENG test battery. They are gaze test, saccade test, positional tests, bithermal caloric test, sinusoidal tracking test, and optokinetic test. In our study, vHIT was preferred because it takes less time, does not cause symptoms such as vertigo and nausea in the patient, and is easier to interpret and apply. The results of the ENG study suggest that there is no significant effect on the acute phase in peripheral vestibular organs assessed by vHIT in patients with AIDS suggesting that the effect may be present in the chronic phase. It may be possible to make further assessments in this area to understand whether the likelihood of this chronic event is due to the direct infection or side effects of the drugs used.

Hausler et al^6^ and Castello et al^7^ discussed that vestibular manifestations may occur due to neurological complications in HIV-infected individuals. Teggi et al^39^ reported that the prevalence of central vestibular involvement was 3.3% in the early stages of the disease and 100% in the advanced stages in their study of 60 HIV-infected patients. In studies evaluating peripheral vestibular disorders, vestibular problems were seen in 1/3 of patients in early stage, and this rate increased to 50% as the disease progressed.^6,7,39^ Acquired immune deficiency syndrome -related opportunist infections and medical treatments that may be toxic to the inner ear used in the treatment of this disease may cause or contribute to vestibular symptoms.^5^ In the current study, even though we did not detect any pathology in SCCs with vHIT in HIV RNA-positive individuals who had not yet been treated, patients who received medical treatment were found to be affected, although not statistically significant. As with the literature results above, the results of this study suggest that increasing vestibular pathologies may be the result of medical treatments preferred for HIV. There are different medicines with different mechanisms of action in current AIDS treatment. Different drugs may be preferred depending on the preference of the physician or course of the disease. It may be useful to conduct vestibular tests on different groups of patients who have different medicines. When we wanted to evaluate the groups separately according to the treatment protocols used in the current study, it was seen that the number of cases is not enough for this. Further studies by creating groups with more patients will be helpful in this regard.

In some studies, it was reported that the subjects that had central neurological involvement and the peripheral vestibular system were not affected.^7^ The post-mortem study by Hart et al^8^ supports this thesis, whereas some post-mortem studies that contradict these reports have reported histopathological changes in the vestibular labyrinth among patients who died from AIDS complications.^40,41^ These conflicting results show that there is a need for additional studies on peripheral vestibular system involvement in patients who have AIDS.

We performed our study with vHIT because of its ability to evaluate the peripheral vestibular system. When vestibular symptoms were questioned in our study, there were no vestibular complaints in individuals who were in the active phase with high HIV RNA and had not received any treatment yet or who had received medical treatment and the HIV RNA level became negative. In addition, none of our cases had signs of hearing loss, tinnitus, or other symptoms that could indicate that the inner ear was affected. As a result of the current study, when all groups were compared, more O/C saccades were detected in the RP canal in the group treated after diagnosis and had negative HIV RNA. Only pathology in 1 canal may be due to a low number of patients. For this reason, it may be useful to repeat the study with groups that have a higher number of volunteers.

In conclusion, video head impulse test is a newly developed test that is easy to apply, does not take much time, is very safe, and has acceptable sensitivity for detecting peripheral vestibular lesions.

The etiology of vestibular dysfunction associated with AIDS is complex. In patients with AIDS, there may be a direct or indirect effect on the peripheral and central vestibular system. Human immunodeficiency virus-infected individuals now have a longer life expectancy due to positive developments in treatment. However, quality of life can be reduced by vestibular symptoms such as vertigo and dizziness.

In this study, it was determined that SCCs were not affected in the patients who had positive HIV RNA and were not treated yet. Pathological findings were determined only in the patients who were treated with medications and had negative HIV RNA. The authors of this study consider that vestibulopathologic findings may be related to the used medications. It may be beneficial to perform further study with more patients who receive medications.

## Figures and Tables

**Table 1.** The Age and Gender Evaluation In-Between Groups

	Group		
A	B	C
**Age,** med ± SD	32.12 ± 7.93	39.64 ± 10.96	36.42 ± 9.93
**Gender,** n (%)			
**Women**	0 (0%)	3 (13.6%)	3 (12.5%)
**Men**	26 (100%)	19 (86.4%)	21 (87.5%)

One-way anova test; Chi square test; *P* < .05.

Med, median; SD, standard deviation.

**Table 2.** The Minimum, Maximum, Average, and Standard Deviation Values of the Research Parameters

Group		Min-Max	Ave ± SS
**Group A** **(HIV RNA+)**	**HIV RNA level**	129-1.6 × 10^7^	1.6 × 10^6^ ± 3.5 × 10^6^
	**HIV RNA level**	129-16411225	8.9 × 10^4^ (median), 1.4 × 10^4^ (quartile deviation)
	**CD4+ level**	20-1102	349.38 ± 257.18
	**LL**	0.82-1.04	0.91 ± 0.05
	**RL**	0.85-1.28	0.97 ± 0.09
	**LA**	0.5-1.08	0.86 ± 0.14
	**RP**	0.58-1.02	0.79 ± 0.11
	**LP**	0.68-0.96	0.79 ± 0.08
**RA**	0.69-0.99	0.84 ± 0.09
**Group B** **(HIV RNA**−**/anti-HIV+)**	**HIV RNA level**	0-0	0 ± 0
	**CD4+ level**	207-1549	682.41 ± 382.84
	**LL**	0.8-1.32	0.92 ± 0.11
	**RL**	0.83-1.18	0.97 ± 0.1
	**LA**	0.77-1.04	0.86 ± 0.07
	**RP**	0.59-0.99	0.75 ± 0.09
	**LP**	0.68-0.92	0.81 ± 0.07
**RA**	0.75-1.19	0.94 ± 0.12
**Group C** **(healthy check)**	**LL**	0.79-1.05	0.91 ± 0.07
	**RL**	0.87-1.29	0.98 ± 0.11
	**LA**	0.68-0.99	0.83 ± 0.09
	**RP**	0.39-0.9	0.72 ± 0.12
	**LP**	0.59-0.95	0.78 ± 0.1
**RA**	0.73-1.08	0.89 ± 0.11

LL, left lateral semicircular canal; RL, right lateral semicircular canal; LA, left anterior semicircular canal; RP, right posterior semicircular canal; LP, left posterior semicircular canal; RA, right anterior semicircular canal; HIV, human immunodeficiency virus.

**Table 3.** The Dispersion of LL, RL, LA, RP, LP, and RA Parameters In-Between Groups with Respect to the Existence of the Decrease in VOR Gain

Group	Decrease in VOR Gain		n	%
**Group A** HIV RNA+ (n = 24)	**LL**	**Pos(+)**	0	0
	**Neg(**−**)**	24	100
	**RL**	**Pos(+)**	0	0
	**Neg(**−**)**	24	100
	**LA**	**Pos(+)**	3	12.5
	**Neg(**−**)**	21	87.5
	**RP**	**Pos(+)**	3	12.5
	**Neg(**−**)**	21	87.5
	**LP**	**Pos(+)**	2	8.3
	**Neg(**−**)**	22	91.7
	**RA**	**Pos(+)**	1	4.2
**Neg(**−**)**	23	95.8
**Group B** **HIV RNA-/anti-HIV+**	LL (n = 21)	**Pos(+)**	0	0
	Neg(−)	21	100
	RL (n = 21)	**Pos(+)**	0	0
	Neg(−)	21	100
	LA (n = 21)	**Pos(+)**	0	0
	Neg(−)	21	100
	RP (n = 21)	**Pos(+)**	6	28.6
	Neg(−)	15	71.4
	LP (n = 21)	**Pos(+)**	1	4.8
	Neg(−)	20	95.2
	RA (n = 20)	**Pos(+)**	0	0
Neg(−)	20	100
**Group C** Healthy (n = 22)	**LL**	**Pos(+)**	1	4.5
	Neg(−)	21	95.5
	**RL**	**Pos(+)**	0	0
	Neg(−)	22	100
	**LA**	**Pos(+)**	2	9.1
	Neg(−)	20	90.9
	**RP**	**Pos(+)**	8	36.4
	Neg(−)	14	63.6
	**LP**	**Pos(+)**	3	13.6
	Neg(−)	19	86.4
	**RA**	**Pos(+)**	0	0
Neg(−)	22	100

LL, left lateral semicircular canal; RL, right lateral semicircular canal; LA, left anterior semicircular canal; RP, right posterior semicircular canal; LP, left posterior semicircular canal; RA, right anterior semicircular canal; HIV, human immunodeficiency virus; VOR, vestibulo-ocular reflex.

**Table 4.** The Dispersion of LL, RL, LA, RP, LP, and RA Parameters in Groups with Respect to O/C Saccade Existence

Group	O/C Saccade Existence		n	%
**Group A** HIV RNA+ (n = 24)	**LL**	**Pos(+)**	0	0
	Neg(−)	24	100
	**RL**	**Pos(+)**	0	0
	Neg(−)	24	100
	**LA**	**Pos(+)**	0	0
	Neg(−)	24	100
	**RP**	**Pos(+)**	0	0
	Neg(−)	24	100
	**LP**	**Pos(+)**	0	0
	Neg(−)	24	100
	**RA**	**Pos(+)**	0	0
Neg(−)	24	100
**Group B** HIV RNA−/anti HIV+	LL (n = 21)	**Pos(+)**	1	4.8
	Neg(−)	20	95.2
	RL (n = 21)	**Pos(+)**	1	4.8
	Neg(−)	20	95.2
	LA (n = 21)	**Pos(+)**	0	0
	Neg(−)	21	100
	RP (n = 21)	**Pos(+)**	2	9.5
	Neg(−)	19	90.5
	LP (n = 21)	Pos(−)	3	14.3
	Neg(−)	18	85.7
	RA (n = 20)	**Pos(+)**	0	0
Neg(−)	20	100
**Group C** Healthy (n = 22)	**LL**	**Pos(+)**	0	0
	Neg(−)	22	100
	**RL**	**Pos(+)**	1	4.5
	Neg(−)	21	95.5
	**LA**	**Pos(+)**	0	0
	Neg(−)	22	100
	**RP**	**Pos(+)**	2	9.1
	Neg(−)	20	90.9
	**LP**	**Pos(+)**	0	0
	Neg(−)	22	100
	**RA**	**Pos(+)**	0	0
Neg(−)	22	100

LL, left lateral semicircular canal; RL, right lateral semicircular canal; LA, left anterior semicircular canal; RP, right posterior semicircular canal; LP, left posterior semicircular canal; RA, right anterior semicircular canal; HIV, human immunodeficiency virus; O, overt; C, covert.

**Table 5.** The Assessment of LL, RL, LA, RP, LP, and RA Parameters in Groups with Respect to the Existence of the Decrease in VOR Gain

VOR Gain		Group A n (%)	Group B n (%)	Group C n (%)	*P*
**LL**	**Pos(+)**	0 (0)	0 (0)	1 (4.5)	**1** **.642**
Neg(−)	24 (100)	21 (100)	21 (95.5)
**RL**	**Pos(+)**	0 (0)	0 (0)	0 (0)	*
Neg(−)	24 (100)	21 (100)	22 (100)
**LA**	**Pos(+)**	3 (12.5)	0 (0)	2 (9.1)	**1** **.359**
Neg(−)	21 (87.5)	21 (100)	20 (90.9)
**RP**	**Pos(+)**	3 (12.5)	6 (28,6)	8 (36.4)	**2** **.164**
Neg(−)	21 (87.5)	15 (71.4)	14 (63.6)
**LP**	**Pos(+)**	2 (8.3)	1 (4,8)	3 (13.6)	**1** **.674**
Neg(−)	22 (91.7)	20 (95,2)	19 (86.4)
**RA**	**Pos(+)**	1 (4.2)	0 (0)	0 (0)	**1** **1.000**
Neg(−)	23 (95.8)	20 (100)	22 (100)

^1^Fisher Hamilton test; ^2^Chi-square test.

*No comparison could be made for all values were 0 between 3 groups.

LL, left lateral semicircular canal; RL, right lateral semicircular canal; LA, left anterior semicircular canal; RP, right posterior semicircular canal; LP, left posterior semicircular canal; RA, right anterior semicircular canal; VOR, vestibulo-ocular reflex.

**Table 6.** The Evaluation of LL, RL, LA, RP, LP, and RA Parameters in Groups with Respect to O/C Saccade Existence

O/C Saccade Existence		Group A n (%)	Group B n (%)	Group C n (%)	*P*
**LL**	**Pos(+)**	0 (0)	1 (4.8)	0 (0)	**.313**
Neg(−)	24 (100)	20 (95.2)	22 (100)
**RL**	**Pos(+)**	0 (0)	1 (4.8)	1 (4.5)	**.533**
Neg(−)	24 (100)	20 (95.2)	21 (95.5)
**LA**	**Pos(+)**	0 (0)	0 (0)	0 (0)	*
Neg(−)	24 (100)	21 (100)	22 (100)
**RP**	**Pos(+)**	0 (0)	2 (9.5)	2 (9.1)	**.378**
Neg(−)	24 (100)	19 (90.5)	20 (90.9)
**LP**	**Pos(+)**	0 (0)	3 (14.3)	0 (0)	.028*
Neg(−)	24 (100)	18 (85.7)	22 (100)
**RA**	**Pos(+)**	0 (0)	0 (0)	0 (0)	
Neg(−)	24 (100)	20 (100)	22 (100)

Fisher Hamilton test was used for LL, LA, LP, RA; Chi Square test was used for RP; * *P* < .05.

*No comparison could be made for all values were 0 between 3 groups.

LL, left lateral semicircular canal; RL, right lateral semicircular canal; LA, left anterior semicircular canal; RP, right posterior semicircular canal; LP, left posterior semicircular canal; RA, right anterior semicircular canal; O, overt; C, covert.
